# The Transcription Factor AmrZ Utilizes Multiple DNA Binding Modes to Recognize Activator and Repressor Sequences of *Pseudomonas aeruginosa* Virulence Genes

**DOI:** 10.1371/journal.ppat.1002648

**Published:** 2012-04-12

**Authors:** Edward E. Pryor, Elizabeth A. Waligora, Binjie Xu, Sheri Dellos-Nolan, Daniel J. Wozniak, Thomas Hollis

**Affiliations:** 1 Department of Biochemistry and Center for Structural Biology, Wake Forest School of Medicine, Winston-Salem, North Carolina, United States of America; 2 Department of Microbiology and Immunology, Wake Forest School of Medicine, Winston-Salem, North Carolina, United States of America; 3 Departments of Microbiology and Microbial Infection and Immunity, Center for Microbial Interface Biology, The Ohio State University, Columbus, Ohio, United States of America; Yale University School of Medicine, United States of America

## Abstract

AmrZ, a member of the Ribbon-Helix-Helix family of DNA binding proteins, functions as both a transcriptional activator and repressor of multiple genes encoding *Pseudomonas aeruginosa* virulence factors. The expression of these virulence factors leads to chronic and sustained infections associated with worsening prognosis. In this study, we present the X-ray crystal structure of AmrZ in complex with DNA containing the repressor site, *amrZ1*. Binding of AmrZ to this site leads to auto-repression. AmrZ binds this DNA sequence as a dimer-of-dimers, and makes specific base contacts to two half sites, separated by a five base pair linker region. Analysis of the linker region shows a narrowing of the minor groove, causing significant distortions. AmrZ binding assays utilizing sequences containing variations in this linker region reveals that secondary structure of the DNA, conferred by the sequence of this region, is an important determinant in binding affinity. The results from these experiments allow for the creation of a model where both intrinsic structure of the DNA and specific nucleotide recognition are absolutely necessary for binding of the protein. We also examined AmrZ binding to the *algD* promoter, which results in activation of the alginate exopolysaccharide biosynthetic operon, and found the protein utilizes different interactions with this site. Finally, we tested the *in vivo* effects of this differential binding by switching the AmrZ binding site at *algD*, where it acts as an activator, for a repressor binding sequence and show that differences in binding alone do not affect transcriptional regulation.

## Author Summary

The bacterium *Pseudomonas aeruginosa* causes a variety of human infections and is the leading cause of death in patients with cystic fibrosis. The main reason for the severity of these infections arises from the ability of *P. aeruginosa* to express virulence factors that protect it from the host immune system. Several of these processes are controlled by a transcription factor called AmrZ, a potential target for anti-microbial therapeutics. AmrZ is unusual in that it has the ability to both activate some genes, such as for alginate biofilm, and repress others, as with flagellum and itself. Here we determine the three dimensional structure of AmrZ bound to DNA containing a repressor sequence. Our structure shows the specific interactions the protein makes with the DNA for binding and repression. It also reveals that both the sequence and shape of the DNA are important for tight association. We next examined the binding of the protein to DNA containing an activator sequence and found that it has different interactions. However, by switching the AmrZ binding site at *algD*, where it acts as an activator, for a repressor binding sequence in *P. aeruginosa*, we show that differences in binding alone do not account for transcriptional regulation.

## Introduction

*Pseudomonas aeruginosa* is an opportunistic, Gram negative bacterium that causes a variety of infections, mainly in immune-challenged patients [1]–[3]. More notably, chronic lung infection by *P. aeruginosa* is the leading cause of death in patients with the autosomal recessive disorder cystic fibrosis (CF) [4]. The underlying cause of the severity of these infections is due in part to the arsenal of virulence factors *P. aeruginosa* has at its disposal, including type III secretion systems, production of biofilms, phospholipase, exotoxin A, motility, and lipopolysaccharide. In alginate producing strains isolated from CF patients, the transcription factor AmrZ (Alginate and Motility Regulator Z, formerly AlgZ) is highly expressed [5]. Our previous work has shown AmrZ functions as both a transcriptional activator and repressor of several virulence factors. AmrZ is necessary for alginate production, via the activation of *algD*, which is the first gene in the alginate biosynthetic operon [6]. Reciprocal to this, AmrZ represses *fleQ*, which encodes an activator of flagellum expression [7]. AmrZ is also required for the regulation of genes responsible for type IV pili localization and twitching motility, through the interaction with a currently unknown gene target [8]. Finally, AmrZ also represses its own transcription by binding to two sites on the *amrZ* promoter, *amrZ1* and *amrZ2* [9].

The 108 amino acid, 12.3 kD AmrZ protein is a member of the ribbon-helix-helix (RHH) family of DNA binding proteins, sharing highest sequence similarity to the Arc and Mnt repressors from bacteriophage P22 [10]. Sequence analyses predict that there are over 2300 proteins containing RHH domains found in bacteria, Archaea, and bacteriophages; however, less than twenty of these proteins have been studied with structural or biochemical techniques [11]. Structural information from RHH proteins both in the presence [12]–[19] and absence [20]–[27] of operator DNA, show that they exist as dimers, formed by a hydrophobic core created by the two α-helices. The majority of RHH proteins are transcriptional repressors. AmrZ and *Helicobacter pylori* NikR are currently the only characterized RHH proteins known to function as both transcriptional activators and repressors [23]. DNA binding by RHH proteins occurs by the insertion of the anti-parallel β-sheet formed by one β-strand from each monomer into the major groove of DNA. The interactions between the protein and the recognition site are very specific, and mutations to either the DNA binding β-sheet, or the operator site often have a negative effect on DNA binding [27], [28]. In addition to binding DNA as a dimer, RHH proteins also assemble as tetramers, which are stabilized by other domains in the protein, such as occurs with the C-terminal domain of Mnt [29].

Information from sequence alignments and structural predictions define three regions of the AmrZ protein, an extended N-terminus spanning residues 1–16, the RHH domain, located from residues 13–66, and a C-terminal domain from residues 67–108 [30]. Both the extended N-terminus and the C-terminal domain do not share any sequence similarity to other proteins, and their exact function has remained an open question. The extended N-terminus has been hypothesized to play a role in DNA binding, and is conserved in other AmrZ orthologs of *P. putida* and *P. syringae* [31]. Extended N-termini of other RHH proteins have been examined, although their functions vary between cofactor binding, oligomerization and protein-protein interactions, ATP hydrolysis, in addition to having roles in DNA recognition. The C-terminal domain of AmrZ is proposed to be involved in protein oligomerization, which is supported by glutaraldehyde cross linking assays that show AmrZ forms oligomeric species consistent with the molecular weight of dimers and tetramers in solution [30].

Of the genes that are regulated by AmrZ, the specific locations of the binding sites are only known for two of them. AmrZ functions as a transcriptional activator at the *algD* promoter and binds 282 base pairs upstream of the transcriptional start site. Additionally, AmrZ acts as a transcriptional repressor of its own gene and recognizes two sites on the *amrZ* promoter (*amrZ1* and *amrZ2*) at positions −93 and −161. Interestingly, DNA foot-printing has been performed at each of these three sites, and little sequence consensus is shared among them [6], [9]. Both the *algD* operon and *amrZ* are under the control of the alternative sigma factor AlgT (AlgU/σ^22^) [32]. Expression of the *algD* operon requires additional factors, including the response regulators AlgB and AlgR, the nucleosome proteins IHF and AlgP, and the AlgQ protein, each being necessary, but not sufficient to activate transcription on their own [5]. To date, only the AmrZ and AlgT proteins are known to interact with the *amrZ* promoter region.

Open questions have remained as to the exact strategies employed by AmrZ to function as both a transcriptional activator and repressor. It is unclear what specific interactions the protein makes with both activator and repressor sequences within DNA in order to carry out these functions. To answer these questions, we determined the crystal structure of an AmrZ C-terminal truncation mutant, Δ42 AmrZ, in complex with an 18 bp oligonucleotide containing the *amrZ1*-binding site. This structure defines the specific recognition site as two half sites separated by a linker region, and provides evidence that the extended N-terminus of AmrZ interacts with the DNA in a sequence independent manner. Site directed mutagenesis experiments of the *amrZ1* DNA reveal that recognition is not only based on the direct readout of the nucleotide sequences, but also relies on recognition of the intrinsic shape of the DNA. These data allow for the creation of a model for transcriptional repression by AmrZ, where a combination of specific base recognition at two half sites and recognition of intrinsic DNA structure allow for binding. We also examined the interaction of AmrZ with the *algD*-binding site, where AmrZ binding functions as an activator of alginate biosynthesis. The results from these assays only identify one AmrZ binding site on *algD* and also suggest that the protein may utilize an additional residue in DNA binding. Finally, we demonstrate that while there are different protein interactions at the activator and repressor sequences, these differences alone do not account for the activator and repressor activity of AmrZ.

## Results/Discussion

### Structural overview of the Δ42 AmrZ: 18 bp amrZ1 complex

**Figure 1:**
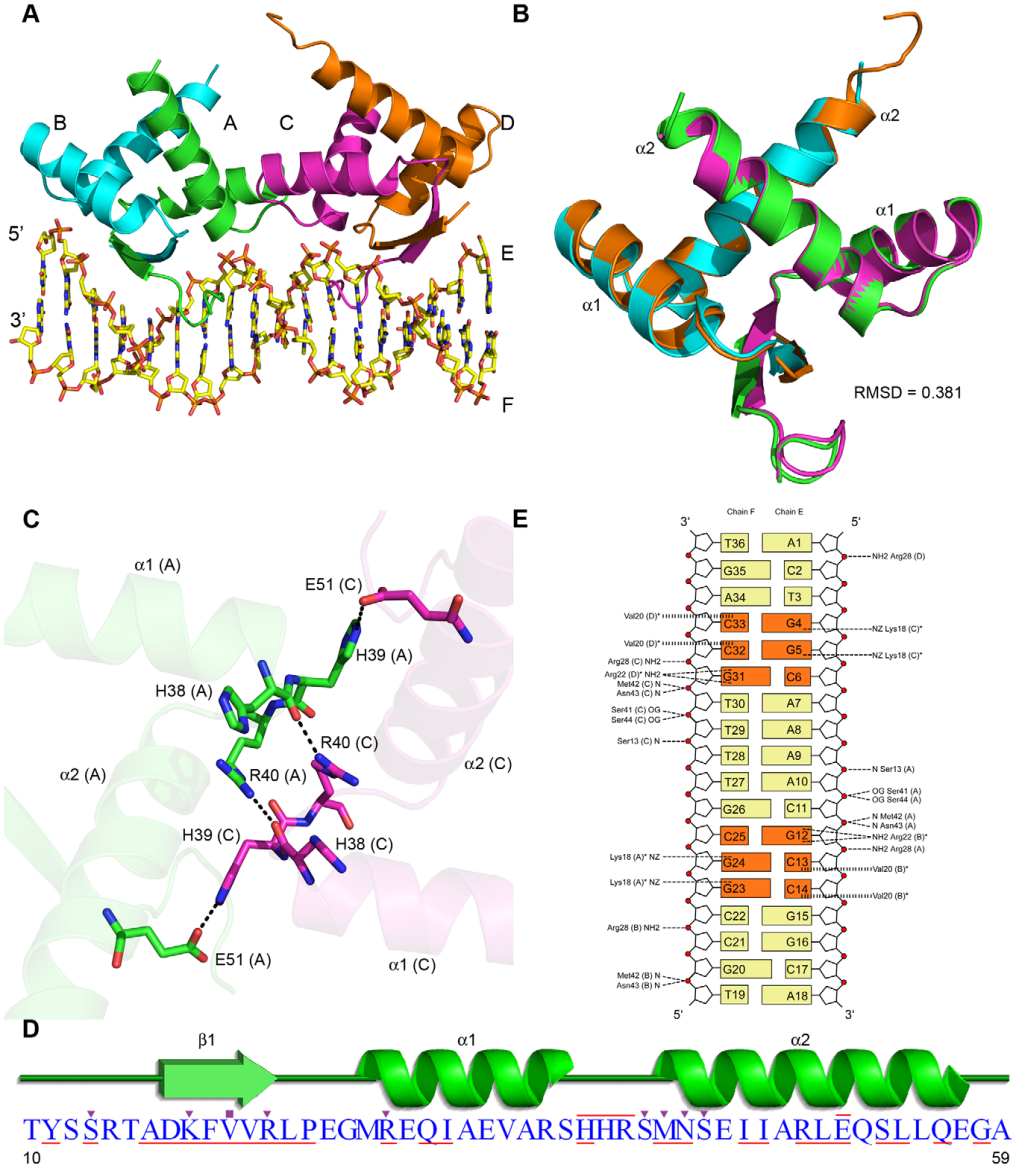
Structural overview of AmrZ - *amrZ1* complex. (A) The Δ42 AmrZ protein binds to the 18 bp *amrZ1* binding site as a dimer of dimers. One dimer is composed of chains A and B (green/cyan), while the other dimer is composed of chains C and D (magenta/orange). (B) The superposition of AmrZ dimers show no major structural differences between them (Cα RMSD = 0.381 Å). (C) The dimer - dimer interface is created by a network of hydrogen bonds between the residues in the loop region between α-helix 1 and α-helix 2 of chains A and C. (D) Secondary structure representation of one AmrZ ribbon-helix-helix monomer. Residues forming hydrogen bonds to DNA are indicated by the purple triangles, while residues forming hydrophobic interactions to DNA are indicated by purple squares. Residues forming the dimer interface between each monomer are underlined in red, and the residues which form the dimer-dimer interface are overlined in red. (E) Schematic of both the sequence dependent and sequence independent interactions between AmrZ and *amrZ1*. Hydrogen bonding interactions to the DNA are illustrated with a short dashed line, while hydrophobic interactions are illustrated with a vertical dashed line. Nucleotides involved in sequence specific interactions are represented in orange. The peptide chain for each residue is labeled in parentheses, and residues that make contacts to more than one nucleotide are notated with an asterisk.

We determined the structure of a C-terminal truncation mutant of AmrZ, Δ42, in complex with an 18 bp oligonucleotide containing the *amrZ1* site to 3.1 Å resolution (Table 1 and Figure 1A). The Δ42 variant of AmrZ (residues 1–66) contains the extended N-terminus and the RHH DNA binding domain, but has a truncated C-terminal domain. Many C-terminal truncation variants of AmrZ were used in crystallization experiments, and Δ42 was the only AmrZ construct tested that crystallized either in the presence or absence of DNA. The Δ42 AmrZ protein was tested for DNA binding affinity, and compared with the wild type protein, no reduction in affinity to any of the three known AmrZ binding sequences (*amrZ1*/*amrZ2*/*algD*) was observed (data not shown). The structure reveals AmrZ binds the *amrZ1* site as a dimer-of-dimers, and DNA recognition occurs by the interaction with two half sites on the DNA, separated by five base pairs. There are no major structural differences between each AmrZ dimer (Cα RMSD = 0.381 Å) (Figure 1B). The dimer-dimer interface, which occludes approximately 290 Å^2^ of surface area on each dimer, is formed by a series of interactions between specific residues located on the loop connecting α-helix 1 and α-helix 2 on chains B and C (Figure 1C). The interactions in this region are symmetric, with the backbone carbonyl of His38 of one protomer forming a hydrogen bond to Arg40 of the opposing protomer, and the side chain of His39 forming a salt bridge to Glu51, also across the interface. The relatively small interface between each dimer, in combination with evidence that AmrZ forms higher order oligomers in solution [30], suggests there are likely additional dimer-dimer interactions mediated by the C-terminal domain of the protein.

**Table 1:** Crystallographic data collection and refinement statistics.

**Data Collection Statistics**	
Wavelength (nm)	0.9793
Spacegroup	I422
Unit Cell Parameters (a, b, c)(Å)	129.5, 129.5, 152.6
(α,β,γ)(°)	90, 90, 90
# Complexes/Asymmetric Unit	1
Unique Reflections	22435
Resolution Range (Å)	40.00 – 3.1 (3.15 – 3.1)
Mean Redundancy	5.7 (5.8)
Overall Completeness (%)	99.7 (100.0)
R_merge_ (%)(a)	6.9 (54.3)
Mean I/σ	29.0 (2.98)
# Se Atoms Found	8
FOM initial	0.41
FOM after density modification	0.65
Model Refinement Statistics	
R_work_ (%)	26.1
R_free_ (%)(b)	29.5
# Protein Atoms	1522
# DNA Atoms	731
# Water Molecules	7
RMSD Bond Angles (°)	0.006
RMSD Bond Lengths (Å)	1.180
Ramachandran Statistics^c^	
Most Favored Regions	153 (89.0%)
Additionally Allowed Regions	19 (11.0%)
Generously Allowed Regions	0 (0.0%)
Disallowed Regions	0 (0.0%)

a Rmerge = (Σ|I−<I>|)/ΣI, where I is the observed intensity and <I> is the average intensity.

b Rfactor = Σ∥F_o_|−|F_c_∥/Σ|F_o_|. Rfree is calculated with the same equation, but with 5% of reflections not used in the refinement.

c Ramachandran statistics are given as the number of amino acids that lie within each region, and the percentage is given in parenthesis.

Values in parenthesis are for the outermost resolution shell (3.15 Å – 3.1 Å).

The interface between AmrZ monomers to form a dimer is primarily composed of α-helix 1 and α-helix 2 of each monomer that come together to form a hydrophobic core. The dimer interface is quite extensive, composed of 25 residues (Figure 1D, underlined residues) and buries approximately 1600 Å^2^ of each monomer. Each AmrZ dimer interacts with the *amrZ1* binding site through sequence dependent interactions (see below), mediated by the insertion of the anti-parallel β-sheet, formed by dimerization, into the major groove of DNA. Additionally, a number of sequence independent interactions to the phosphate backbone are formed, further supporting the protein-DNA complex (Figure 1E). The protein - DNA interactions exclude a total surface area of 1469 Å^2^ and are symmetric on both halves of the DNA. The one exception to this is the α-helix 2 N-terminus of chain D, which is not positioned to interact with the phosphate backbone; however, this is most likely due to the lack of a 5′ phosphate group on the nucleotide A1, an artifact of chemical DNA synthesis.

### Sequence dependent binding by AmrZ

**Figure 2:**
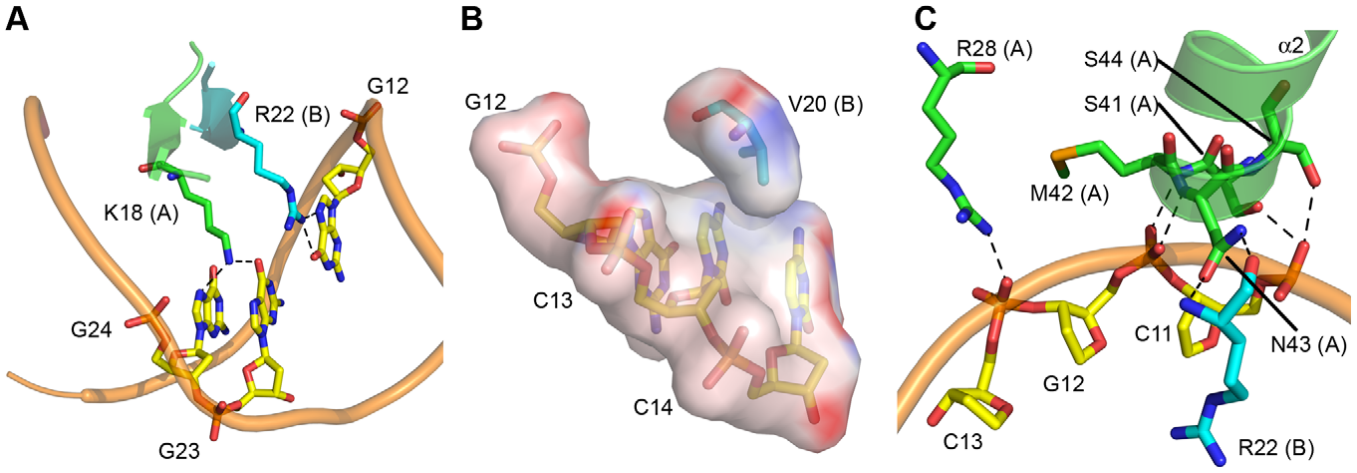
AmrZ - *amrZ1* interactions. (A) Sequence dependent binding by AmrZ is mediated by the insertion of the DNA binding β-sheet into the major groove of DNA. AmrZ recognizes two half sites on the DNA, each with the sequence 5′-GGC. Lys18 is positioned to form hydrogen bonds to multiple O6 and N7 atoms on the adjacent guanine nucleotides, G23 and G24 (G4 and G5 on other half site), while Arg22 forms hydrogen bonds to the O6 and N7 atoms on G12 (G31 on other half site). (B) Electrostatic surface representation of Val20, which is positioned to form hydrophobic interactions to the two nucleotides C13 and C14 (C32 and C33 on other half site). Electropositive surface potential is denoted in blue, while an electronegative surface potential is denoted in red. (C) Sequence independent binding by AmrZ is mediated by the N-terminus of α-helix 2, as well as Arg28 from α-helix 1, which form hydrogen bonding interactions to phosphodiester backbone of multiple nucleotides.

The structure allows us to determine the specific nucleotide sequence recognized by AmrZ, as well as other factors that contribute to DNA recognition. The insertion of the anti-parallel β-sheet from each AmrZ dimer into the major groove of the *amrZ1* site provides for the recognition of two half sites, each with the sequence 5′-GGC (Figure 1E, orange bases). Sequence dependent binding by AmrZ occurs via the interaction of three residues, Lys18, Val20, and Arg22, with the nucleotides. Lys18 from one AmrZ monomer is positioned where it can form hydrogen bonding interactions to the O6 and N7 atoms of the two guanine nucleotides, G23 and G24 (G4 and G5 on the other half site) (Figure 2A). The DNA binding β-sheet also orients the residue Arg22, from the other monomer of the dimer, to form a bidentate hydrogen bond to both the O6 and N7 atoms of the nucleotide G12 (G31 on the other half site). This is on the opposite strand of the two bases with which Lys18 interacts. Bidentate hydrogen bonding, specifically between arginine residues and guanine nucleotides, are a major determinant in the selectivity of DNA bases [33]. Another relevant residue located on the DNA binding β-sheet is Val20. Interestingly, among other RHH proteins, this position in the DNA binding β-sheet is generally conserved as a neutral hydrophilic residue. One other exception to this is the *Neisseria gonorrhoeae* FitAB protein in which there is also a valine at this position [18]. The structure of FitAB in complex with DNA shows the valine forms a van der Waals interaction with the C5 methyl group of a thymine base. In the AmrZ structure, it appears that residue Val20 is poised to select for cytosine bases C13 and C14 (C32 and C33 on other half site) via a hydrophobic interaction (Figure 2B). Purine nucleotides would not be favorable in these locations since the N7 atom of the purine base would interfere with the hydrophobic pocket that is formed by Val20, while a thymine nucleotide in this position would sterically clash with the isopropyl side chain of the valine residue.

To confirm the requirement for each half site in *amrZ1* recognition by AmrZ, a series of mutations were created to the *amrZ1* DNA binding site. Both 1 and 2 nucleotides in each *amrZ1* half site were mutated, and the affinity of WT AmrZ to each of these mutant sequences was measured using fluorescence anisotropy (Table 2). Mutating one nucleotide in each 5′-GGC AmrZ recognition half site to 5′-GTC caused a 9.8 fold reduction in affinity, while mutating two of the nucleotides in each half site to 5′-TTC caused a 12.9 fold reduction in affinity compared to binding to the native *amrZ1* sequence. These results confirm the observations from the structure that sequence dependent recognition occurs through the interactions with two half sites, each with the sequence 5′-GGC.

**Table 2:** AmrZ affinity for *amrZ1* binding site mutants.

Binding Site	Sequence^a^	K_d_ b (nM) ± SE	Fold over WT *amrZ1* c
WT *amrZ1*	GTACTGGCAAAACGCCGGCACG CATGACCGTTTTGCGGCCGTGC	8.41±0.8	1.0
GTC *amrZ1*	GTACTG**T**CAAAACG**A**CGGCACG CATGAC**A**GTTTTGC**T**GCCGTGC	82.7±8.6	9.8
TTC *amrZ1*	GTACT**TT**CAAAACG**AA**GGCACG CATGA**AA**GTTTTGC**TT**CCGTGC	109±13	12.9
CGCG *amrZ1*	GTACTGGC**CGCG**CGCCGGCACG CATGACCG**GCGC**GCGGCCGTGC	63.4±7.3	7.5
TTC/GC *amrZ1*	GTACT**TT**C**CGCG**CG**AA**GGCACG CATGA**AA**G**GCGC**GC**TT**CCGTGC	2050±180	244
TTTT *amrZ1*	GTACTGGC**TTTT**CGCCGGCACG CATGACCG**AAAA**GCGGCCGTGC	41.7±5.1	5.0
AATT *amrZ1*	GTACTGGC**AATT**CGCCGGCACG CATGACCG**TTAA**GCGGCCGTGC	58.6±7.5	7.0
ATAT *amrZ1*	GTACTGGC**ATAT**CGCCGGCACG CATGACCG**TATA**GCGGCCGTGC	39.1±4.6	4.7

a The two AmrZ binding sites on the wild type *amrZ1* sequence are represented by the underlined nucleotides. Mutations to the wild type *amrZ1* binding site are notated by the bolded nucleotides in each mutant sequence.

b The K_d_ was calculated by fitting the hyperbolic equation for a single ligand binding model with saturation (eq 2) to the data in Figure S2, which were averaged from four independent experiments.

c Fold over (wild type) WT *amrZ1* is defined by (K_d_ of sample)/(K_d_ of wild type) for each sample.

We previously evaluated the contribution of Lys18 and Arg22 to AmrZ activity using *in vitro* DNA binding assays at *amrZ1* and transcriptional reporter assays to measure *amrZ* repression [30]. The mutation of Lys18 to an alanine (K18A) resulted in a drastic reduction in the DNA binding activity, causing a 274-fold increase in the dissociation constant (K_d_), compared to WT AmrZ. When *amrZ* was replaced in the *P. aeruginosa* chromosome with a gene encoding K18A AmrZ, *amrZ* derepression was observed, which was similar in magnitude to that observed in strains harboring null *amrZ* alleles. Similar results were obtained for the R22A mutant of AmrZ, which had a 44-fold increase in K_d_ compared to WT AmrZ *in vitro*, and comparable effects of *amrZ* transcription *in vivo*. When the effects of mutating the valine at position 20 to an alanine (V20A) were tested *in vitro*, a 10-fold increase in K_d_ was observed. Even with a smaller reduction in DNA binding ability compared to the K18A and R22A mutants, V20A AmrZ was unable to repress *amrZ* transcription *in vivo*.

### The extended N-terminus of AmrZ

**Figure 3:**
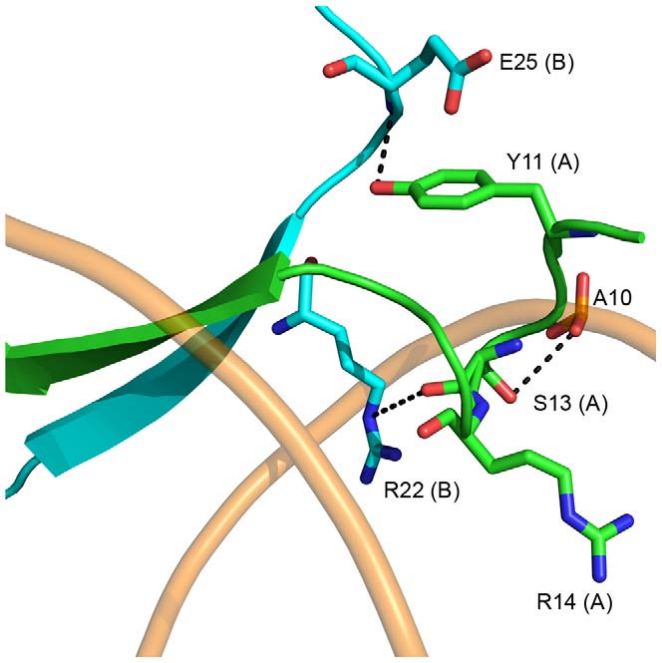
The extended N-terminus of AmrZ. The N-terminus of AmrZ is ordered only when in contact with the DNA (shown as wheat colored tubes). It forms a looped structure which is stabilized by Glu25 from chain B and Tyr11 from chain A. This region positions the side chains of two residues, Ser13 and Arg14 in the major groove of DNA. Ser13 forms hydrogen bonding contacts to the phosphate backbone, while Arg14 is not positioned to contact any nucleotides. This loop also interacts with the DNA binding residue Arg22 via the hydrogen bond between the backbone carbonyl of Ser13 and the εN of the arginine side chain.

We observe electron density for the extended N-terminus starting at residue 10 on chains A and C. This density is only observed on the side of the AmrZ dimer that makes the specific contacts to the *amrZ1* binding site. The lack of electron density of the extended N-terminus on the side of the AmrZ dimer that does not contact the DNA suggests that the N-terminus is disordered in solution, and becomes structured upon DNA binding. Residues 10–17 of the N-terminus form a looped structure, allowing the amino acids Ser13 and Arg14 in the major groove to interact with the DNA (Figure 3). This looped structure is supported by the residue Tyr11, which forms a hydrogen bond to the backbone of Glu25 from the other monomer in the dimer, and by the head-on orientation of the carboxyl side chain of Glu25 perpendicular to the aromatic ring of Tyr11. The side chain of Ser13 forms a hydrogen bond to a phosphate in the DNA backbone, and also positions the residue Arg14 into the major groove of the DNA; however, no contacts between Arg14 and the DNA bases are observed in the structure. This is consistent with previous studies of an AmrZ R14A mutant, which has no change in binding affinity for the *amrZ1* DNA when compared to WT protein *in vitro*, as well as no effect on *amrZ* repression *in vivo* [30]. Other RHH proteins contain extended N-termini that contribute to DNA binding. The *Staphylococcus aureus* pSK41 plasmid-encoded ArtA protein has a 16 residue N-terminal domain that is necessary for recognition of at least one of the binding sites of the protein [19]. Additionally, the seven residue extended N-terminus of the Arc repressor is disordered in solution, but adopts a tandem-turn structure upon binding DNA [13], and mutations to the N-terminus result in decreased binding to operator sites [34]. Although mutations to the extended N-terminus in AmrZ do not reduce affinity to the *amrZ1* repressor site, AmrZ may act in a manner similar to ArtA, where the extended N-terminus may provide specificity for DNA binding at other sites.

### Sequence independent binding

There are a number of interactions between AmrZ and the phosphodiester backbone of the DNA that act to position the DNA binding β-sheet in the major groove. The majority of these sequence independent interactions occur with residues located in the N-terminus of α-helix 2, which points down towards the DNA backbone (Figure 2C). The positioning of this helix allows the formation of hydrogen bonding interactions between the side chains of Ser41 and Ser44, and the backbone amide nitrogens of Met42 and Asn43 to the phosphate groups of the DNA. This interaction is further bolstered by the positive dipole of the N-terminal end of α-helix 2 and the negatively charged phosphate backbone of the DNA. Another sequence independent interaction between AmrZ and the DNA occurs via the side chain of Arg28, from α-helix 1, to the backbone of the DNA. Interestingly, the location of α-helix 2 also allows the side chain of Asn43 to form two hydrogen bonding interactions to the backbone amide nitrogen and carbonyl oxygen of the DNA binding residue, Arg22, in the opposite monomer. This also helps position Arg22 for interaction with the DNA bases. Sequence independent interactions formed by the N-terminus of α-helix 2 are one of the main structural features of RHH proteins [11]. These contacts are often observed to anchor the protein onto the DNA; however, in the case of AmrZ, this electrostatic interaction may play an additional role in recognition of the intrinsic shape of the DNA, particularly in the linker region.

### The linker region between amrZ1 half sites confers specificity

**Figure 4:**
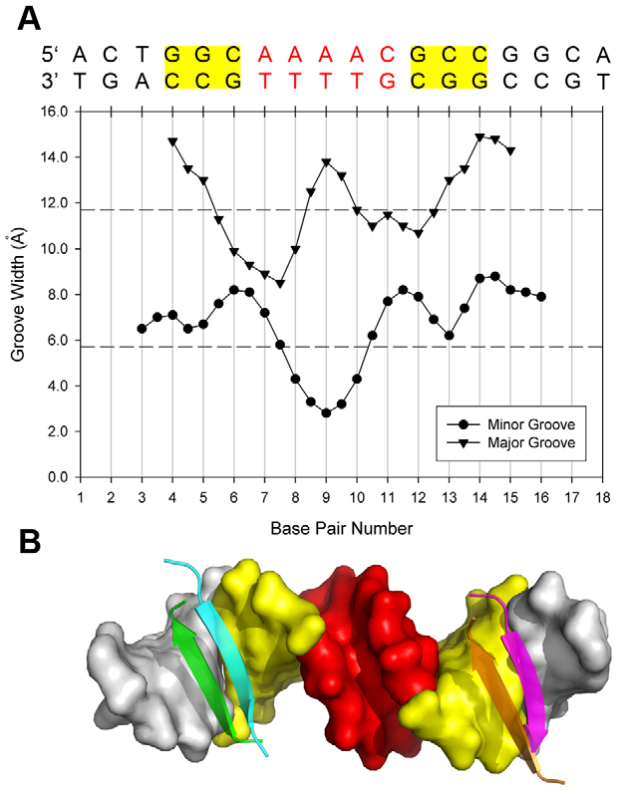
The DNA linker region between the two AmrZ binding half sites has a distorted structure. (A) Major and minor groove widths of the *amrZ1* binding site are plotted on the graph with the corresponding nucleotide sequence above. Values are of inter-phosphate distance minus their van der Waals surface. Also indicated are the average major and minor groove widths of 11.7 Å, and 5.7 Å, respectively, of B-form DNA (horizontal dashed lines) [68]. The minor groove in the region between each AmrZ binding half site is narrowed to 2.8 Å (B) Surface representation of the *amrZ1* DNA. The two AmrZ binding half sites are colored in yellow, while the distorted minor groove is colored in red. Also shown is the position of the DNA binding β-sheets on each binding half site.

Analysis of the *amrZ1* DNA in the structure reveals a significant narrowing of the minor groove to 2.8 Å in the A/T rich region between the two *amrZ1* half sites (Figure 4A). In addition to the narrow minor groove, there is an increase in the width of the major grooves where AmrZ interacts with each half site, most likely to accommodate the width of the anti-parallel β-sheet in this region (Figure 4B). A-tract DNA, as in the *amrZ1* site, has specific properties in that each ApA base pair step exhibits a negative roll, and bifurcated hydrogen bonds between each adenine and two thymine nucleotides on the opposite strand lead to propeller twisting and minor groove narrowing; A-tract DNA is also thought to be less flexible due to the extra stabilization provided by the additional bifurcated hydrogen bonds [35]. Based on this we investigated the region between the two *amrZ1* half sites for any role in AmrZ binding, and whether the binding of AmrZ causes distortions to the *amrZ1* DNA, or if the *amrZ1* DNA is intrinsically distorted, allowing for AmrZ recognition.

To test if the linker region between each AmrZ binding half site contributes to AmrZ affinity at *amrZ1*, the native A/T rich linker sequence 5′-AAAAC was mutated to a G/C rich linker region with the sequence 5′-CGCGC, which resulted in a 7.5 fold reduction in binding (Table 2). It is important to note that this reduction in binding is not caused by the removal of specific protein - nucleotide interactions, since there are no contacts between the AmrZ protein and *amrZ1* DNA in this region. Combining the mutations in the AmrZ binding half site with the mutations to the linker region (TTC/GC *amrZ1*) caused a severe aberration in binding affinity (244-fold reduction). The results show that binding affinity is regulated by both the sequence dependent interactions between AmrZ and *amrZ1* and the linker region separating these binding sites.

Additional binding experiments were performed to determine if the intrinsic structure of the A/T rich linker contributes to binding affinity. The five base pair linker region on the native *amrZ1* binding site was mutated to three sequences, each having their own unique properties. A sequence with the linker region mutated to 5′-TTTTC resulted in a 5.0-fold reduction in AmrZ binding, when compared to the WT *amrZ1* sequence (Table 2). TpT base pair steps have the same properties of ApA base pair steps, including a narrow minor groove and less flexibility [35]. Mutating the *amrZ1* sequence to 5′-AATTC caused a 7.0-fold reduction in affinity when compared to AmrZ binding to the WT *amrZ1* sequence (Table 2). Molecular dynamics simulations of the interactions between the papillomavirus E2 transcription factors and their binding sites have shown that the 4 nucleotide sequence AATT has similar minor groove and propeller twist properties to A-tract DNA [36]. The last *amrZ1* mutant binding site tested had a linker region containing the sequence 5′-ATATC, and AmrZ binding to this site was also altered compared to the WT *amrZ1* sequence, causing a 4.7-fold reduction in affinity (Table 2). This site was designed to test if flexibility in the linker region allowed AmrZ to distort the DNA and form a complex. The TpA step in this sequence permits variations in roll, twist and slide due to poor stacking between these base pairs, and DNA containing these steps contain wider minor grooves, caused by the steric clashing of cross strand adenines [37]. It should be noted that the properties described for these sequences are average parameters derived from structures and that individual structures show a range of properties, specifically minor groove width [38]. Although AmrZ had reduced affinity for each of these three sequences, the most dramatic effect was mutation of the linker region to 5′-CGCGC. Binding of AmrZ to the sequence 5′-ATATC was reduced suggesting that A/T content of the sequence, which is usually thought to impart flexibility to DNA, was not the main contributor to AmrZ specificity. AmrZ binding to the two sequences harboring mutant linker regions with similar properties to the A-tract sequence (5′-TTTTC and 5′-AATTC) was decreased, suggesting that there are properties unique to the 5′-AAAAC linker sequence in the native *amrZ1* binding site that allow for binding specificity. Taken together, these data allow us to propose that binding specificity is directed by intrinsic distortions to the DNA, rather than the flexibility conferred by the A/T rich sequence composition. Recognizing a physical feature of the DNA rather than a specific sequence introduces degeneracy in the recognition sequence that would influence the number of potential recognition sites for AmrZ. We queried the *P. aeruginosa* PAO1 genome [31] for the number of binding sites with the exact *amrZ1* repressor sequence and found 5 sites. If we allow the A/T linker region to be degenerate, the number of potential binding sites increases to 77. Further biological studies will be required to determine how many of these sites function as actual regulators.

Narrowed minor grooves of DNA have a strong correlation between the width and increased electronegative potential of the minor groove [38]. There are many examples of transcription factors that recognize local distortions of the minor groove in addition to sequence specific recognition in both prokaryotic and eukaryotic organisms. The *Listeria monocytogenes* helix-turn-helix (HTH) transcription factor MogR recognizes two half sites on the *flaA* operator site [39]. The minor groove between the two half sites is distorted, and contributes to MogR specificity for this site. Another example is the myocyte enhancer factor-2 (MEF2), a member of the MADS-box superfamily, which recognizes a narrowed minor groove on the consensus sequence to bind and activate transcription [40]. These two examples, in addition to others, use positively charged residues, specifically arginine, to recognize and form contacts with the enhanced electronegative potential of the narrow minor groove [38]. However, there are examples of proteins similar to AmrZ that recognize minor groove shape, but do not make any contacts to the minor groove. The classical example is the bacteriophage 434 repressor recognition of six binding sites on the two operator regions, O_R_ and O_L_, which is greatly modulated by the sequence composition of the central region of these sites [41]. These variations in binding affinities have been shown to be biologically important in directing the lysogenic or lytic fate of bacteriophage 434 [42]. Although the 434 repressor positions an arginine residue near the minor groove, there are no specific contacts by the protein to this region, and mutational analysis shows that this arginine does not contribute to binding affinity [41]. Recently, recognition of the intrinsic structure of narrowed minor grooves has been studied with the DNA bending protein Fis, which is responsible for the compaction of bacterial DNA [43]. An A/T rich (5′-AATTT) narrowed minor groove, located between two Fis binding sites is compressed, allowing for the insertion of two HTH domains into the adjacent major grooves of DNA. Mutations to this narrow minor groove sequence cause changes in binding, with the biggest change occurring by mutating the sequence to a G/C rich sequence (5′-GGCGC). Narrowed minor grooves between binding half sites have been observed in other RHH protein - DNA structures. In the structure of Arc in complex with DNA, the minor groove between half sites is narrowed to 1.2 Å, and the sequence in this region has the sequence 5′-GTGCT [13]. Likewise, in the *Streptococcus sp*. CopG-DNA structure the minor groove is narrowed to 1.9 Å and has the sequence 5′-TTGAG [14]. The DNA in complex with the *inc18* plasmid encoded omega protein has a minor groove width of 2.7 Å, and the A/T rich sequence 5′-AAAT. Also, due to a fortuitous packing arrangement in the crystal, both bound and free DNA were observed, with the free DNA having similar secondary structure as the omega bound DNA [16]. For each of these proteins, the contributions of the linker region between the two half sites to binding have not been determined.

Alterations in the sequence specific half sites, the linker region, or both can modulate affinity for AmrZ to *amrZ1*; however, the exact mechanism by which AmrZ recognizes the distorted structure of the minor groove remains enigmatic. In the Δ42AmrZ-*amrZ1* structure (Figure 1A), there are no positively charged amino acids that contact the minor groove. The extended N-terminus of AmrZ contains an arginine at position 2 which might make these contacts; however, *in vitro* DNA binding assays performed with various N-terminal truncation mutants of AmrZ showed no decrease in binding to *amrZ1* [30]. The phosphate backbone on either side of the narrow minor groove of *amrZ1* is contacted on each side by the N-terminus of α-helix 2 from chains A and C (Figure 1A, 2C). This attraction is most likely enhanced due to the positive dipole formed by the N-terminus of the α-helix and the increased electronegative potential of the narrowed minor groove.

### AmrZ binding to the activator site, algD

**Figure 5:**
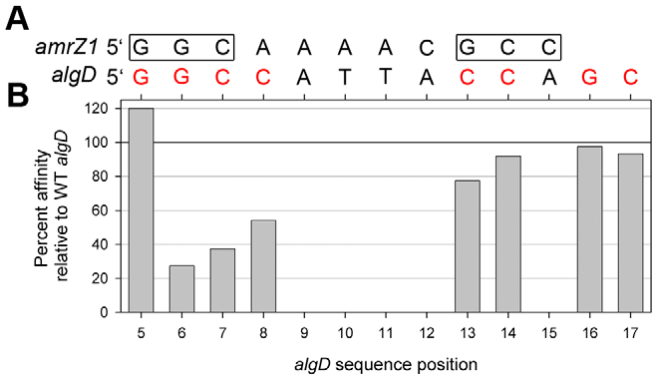
AmrZ binding to the *algD* activator site. (A) Alignment of the *amrZ1* and *algD* DNA sequences that have been derived from previous footprinting experiments [6], [9]. The two binding half sites on the *amrZ1* sequence are boxed, while nucleotides that were mutated in the *algD* sequence are shown in red. (B) Results from the scanning mutagenesis of the *algD* site (Table 3). Only mutations of guanine nucleotides at positions 6 (forward strand), and 7 and 8 (reverse strand) resulted in a noticeable decrease in binding affinity of AmrZ compared to the WT *algD* sequence. Mutations to the right side binding half site resulted in no major decrease in binding affinity of AmrZ. Percent affinity was calculated by dividing the K_d_ for the WT *algD* binding site by the K_d_ for each mutant binding site.

In addition to functioning as a repressor when bound to *amrZ1*, AmrZ binding to the *algD* site is necessary for the activation of genes responsible for alginate biosynthesis. Interestingly, there are significant divergences between the activator and repressor sequences, and AmrZ affinity to the *algD* binding site is approximately 24 fold reduced compared to the *amrZ1* binding site (Tables 2 & 3). Using the information from the AmrZ interaction with the repressor *amrZ1* binding site, we asked if we could predict how AmrZ interacts with the binding site on the *algD* promoter (Figure S1). We set out to determine the features of the *algD* sequence necessary for AmrZ recognition and activation. By aligning the left half AmrZ binding site on *amrZ1* (5′-GGC) to the *algD* sequence (positions 5–7), it became apparent that there is no similar right half binding site on *algD*, and additionally, the sequence of the linker region is also different (Figure 5A). In order to probe the interaction between AmrZ and *algD*, multiple single nucleotide mutations of the *algD* site were created, and binding affinity of AmrZ to each of the mutant *algD* sequences was measured with fluorescence anisotropy.

**Table 3:** AmrZ affinity for *algD* binding site mutants.

Binding Site	Sequence^a^	K_d_ b (nM) ± SE	Fold over WT *algD* c
WT *algD*	CATTGGCCATTACCAGCCTCCC GTAACCGGTAATGGTCGGAGGG	198±14	1.0
m5 *algD*	CATT**T**GCCATTACCAGCCTCCC GTAA**A**CGGTAATGGTCGGAGGG	164±17	0.8
m6 *algD*	CATTG**T**CCATTACCAGCCTCCC GTAAC**A**GGTAATGGTCGGAGGG	723±37	3.7
m7 *algD*	CATTGG**A**CATTACCAGCCTCCC GTAACC**T**GTAATGGTCGGAGGG	530±52	2.7
m8 *algD*	CATTGGC**A**ATTACCAGCCTCCC GTAACCG**T**TAATGGTCGGAGGG	365±30	1.8
m13 *algD*	CATTGGCCATTA**A**CAGCCTCCC GTAACCGGTAAT**T**GTCGGAGGG	256±20	1.3
m14 *algD*	CATTGGCCATTAC**A**AGCCTCCC GTAACCGGTAATG**T**TCGGAGGG	216±17	1.1
m16 *algD*	CATTGGCCATTACCA**T**CCTCCC GTAACCGGTAATGGT**A**GGAGGG	203±17	1.0
m17 *algD*	CATTGGCCATTACCAG**A**CTCCC GTAACCGGTAATGGTC**T**GAGGG	212±19	1.1

a Mutations to the wild type *algD* binding site are notated by the bolded nucleotides in each mutant sequence.

b The K_d_ was calculated by fitting the hyperbolic equation for a single ligand binding model with saturation (eq 2) to the data in Figure S3, which were averaged from four independent experiments.

c Fold over (wild type) WT *algD* is defined by (K_d_ of sample)/(K_d_ of wild type) for each sample.

Through mutagenesis of nucleotides in the proposed left half binding site in *algD*, we show that AmrZ recognizes the sequence 5′-GGC at this site. The guanine nucleotides at positions 5 and 6 on one strand of the *algD* binding site and positions 7 and 8 on the other strand (Figure 5A) were mutated to thymine bases, and the binding affinity of AmrZ to each of these mutant sequences was measured (Table 3, Figure 5B). The mutation to position 5 resulted in a slight increase to AmrZ affinity, while mutations to positions 6, 7, and 8 each resulted in significant reductions in affinity. To determine the nucleotides AmrZ interacts with on the right half of the *algD* binding site, guanine bases at position 16 on one strand, and positions 13, 14, and 17 on the opposite strand of *algD* were mutated to thymine residues. No significant differences in binding affinity to these sequences are observed (Table 3, Figure 5B), suggesting that AmrZ interacts with this half site in a different manner than what is observed at *amrZ1*.

Our previous binding experiments show the same residues, Lys18, Val20, and Arg22 are involved in the sequence dependent interactions with *algD* [30]. In addition, we found that Arg14 is also necessary for binding, with the R14A mutant of AmrZ exhibiting a 5 fold reduction in binding affinity at *algD*. This arginine residue is also required for transcriptional activation of *algD*, where R14A AmrZ only retains 3% of WT activity *in vivo*. From the AmrZ - *amrZ1* structure, the extended N-terminus forms a looped structure which positions Arg14 into the major groove of DNA (Figure 3); however, no specific contacts between this residue and the *amrZ1* DNA are observed, and mutations of this residue have no effects on *in vitro* and *in vivo* activity at the repressor site.

### algD promoter swapping

The differences we observe in interactions of AmrZ with the activator *algD* binding sequence versus the repressor *amrZ1* binding sequence led us to ask if these different binding modes alone could account for activation or repression activity. To test this hypothesis we switched the AmrZ binding site in the *algD* promoter (activator) with the *amrZ1* binding site (repressor) and introduced this variant into an *algD*::*lacZ* transcriptional fusion, which was stably integrated into the genome of the mucoid *P. aeruginosa* strain FRD1 (FRD1 *palgD_amrZ1_-lacZ*). The position and length of the switched binding site were the same as in the native *algD* promoter. With this construct we measured relative activation at *algD* with a β-galactosidase activity assay compared to an *algD*::*lacZ* transcriptional fusion containing the wild type *algD* AmrZ binding site (FRD1 *palgD-lacZ*). The results of this experiment (Table 4) reveal activation of *algD* remains unchanged when the *amrZ1* binding site replaced the native site. Cell lysates from the FRD1 *palgD_amrZ1_-lacZ* strain had 527.7 units of β-galactosidase activity compared to 536.1 units for FRD1 *palgD-lacZ*. Expression of both *palgD-lacZ* and *palgD_amrZ1_-lacZ* were significantly reduced in *amrZ* mutant *P. aeruginosa* strains (Table 4), indicating that the reporter fusion faithfully reproduced what has been observed previously regarding AmrZ activation of *algD* [5], [10], [30]. The activation of *algD* with the *amrZ1* repressor site at its promoter supports a model in which AmrZ binding alone does not regulate activation or repression of transcription, but rather interactions of AmrZ with other regulators at the *amrZ* and *algD* promoters likely contribute to repression or activation, respectively. This is consistent with the previous evidence that the AlgB, AlgR, IHF, and CysB regulators are known to bind on the *algD* promoter and are necessary for activation [44]–[48], suggesting a possible interaction of AmrZ with one of these proteins. To date, no other regulators have been identified to bind the *amrZ* promoter; however, it is possible one of these same regulators may also interact with AmrZ there as well. An additional determinant likely dictating activation versus repression is the position of the AmrZ binding site relative to the start of transcription, which differs for *algD* (−282) and *amrZ1* (−93).

**Table 4:** Transcriptional activation of *algD* containing *amrZ1*-repressor binding sequence.

*P. aeruginosa* Genotype	Units β-galactosidase
FRD1 p*algD-lacZ*	536.1+/−20.5
FRD1 *lacZ*	73.9+/−41.3
FRD1 p*algD_amrZ_-lacZ*	527.7+/−11.6
FRD1 *ΔamrZ* p*algD-lacZ*	114.7+/−15.3
FRD1 *ΔamrZ lacZ*	72.2+/−34.3
FRD1 *ΔamrZ* p*algD_amrZ_-lacZ*	151.3+/−24.4

### Conclusions

AmrZ functions as both a transcriptional activator and repressor of *P. aeruginosa* virulence genes. We have determined the structure of Δ42 AmrZ in complex with an 18 base pair oligonucleotide containing the *amrZ1* binding site. AmrZ binding to this site results in the repression of *amrZ* transcription. By combining structural and biochemical data, we developed a model for AmrZ recognition at *amrZ1*. Using the suggested terminology from the recent review by Rohs et al. [49], the protein-DNA specificity of AmrZ can be classified by major groove base readout through protein residues in the β-sheet with two GGC half sites in the DNA. This is combined with local shape readout utilizing minor groove distortions in the linker region between the half sites. We also probed the interaction of AmrZ with another biologically important binding site *algD*, which leads to the activation of alginate biosynthesis. In contrast, we observed stark differences in the physical interactions that AmrZ makes with the *algD* sequence that suggest the protein likely utilizes a different mode of recognition at this site. AmrZ binds the *algD* sequence with lower affinity, and mutagenesis of the *algD* sequence shows that only one half site contributes to AmrZ binding. However, these differences in protein binding at the promoter sequences are alone not sufficient to account for the activator or repressor activity of AmrZ, and likely the position of AmrZ binding at the promoter and/or protein interactions with other regulators are also necessary for biological function.

## Materials and Methods

### Molecular cloning, expression and purification of WT and Δ42 *P. aeruginosa* AmrZ

The gene encoding WT AmrZ was PCR amplified from the *P. aeruginosa* strain PAO1 with the primers *amrZ_F* (5′-CGCCATCACATATGCGCCCACTGAAACAGGC) and *amrZ_wt_R* (5′-CGCCATCAGGATCCTCAGGCCTGGGCCAGCTC). The resulting gene product was then inserted into a modified pET19 expression vector (Novagen) which encodes an N-terminal poly-Histidine tag, followed by a Rhinovirus 3C protease cleavage site, which permits the removal of the affinity tag (PreScission Protease, GE Healthcare). The pET19-*amrZ* vector was transformed into *E. coli* C41(DE3) cells for expression. One liter of LB-Broth (Luria-Bertani) supplemented with 50 µg/ml of ampicillin was inoculated with 10 ml of an overnight culture of the C41 cells containing the pET19-*amrZ* vector. The cells were grown at 37°C to an OD_600_ = 0.5, and induced with 1 mM isopropyl β-D-thiogalactopyranoside (IPTG) at 16°C for 20 hours. Prior to induction with IPTG, cells were rapidly cooled on ice to 20°C to bring the temperature of the culture close to the induction temperature. Induction of the cells at low temperature was necessary for protein solubility during overexpression. Cells were harvested by centrifugation, resuspended in lysis buffer (100 mM KH_2_PO_4_ pH 7.5, 500 mM NaCl, 10% glycerol, 4 M urea), and lysed using an EmulsiFlex C-5 cell homogenizer (Avestin). Cell debris was removed at 30,000× g and the supernatant was passed over a 10 ml Ni-NTA (Qiagen) column equilibrated with lysis buffer. This column was washed with 20 column volumes of wash buffer 1 (100 mM KH_2_PO_4_ pH 7.5, 500 mM NaCl, 10% glycerol, 35 mM imidazole, 3 M urea), followed by 10 column volumes of wash buffer 2 (100 mM KH_2_PO_4_ pH 7.5, 500 mM NaCl, 10% glycerol, 50 mM imidazole, 2 M urea). Bound AmrZ was eluted with elution buffer (100 mM KH_2_PO_4_ pH 7.5, 500 mM NaCl, 10% glycerol, 500 mM imidazole, 1 M urea), treated with PreScission Protease according to the manufacturer's directions, and dialyzed over night at 4°C against 100 mM Bis-Tris pH 5.5, 100 mM NaCl, 5% glycerol, 2 mM dithiothreitol (DTT), and 0.5 mM EDTA. The partial denaturing conditions introduced by the 4 M urea were necessary for protein solubility and affinity to the Ni-NTA column, and no change in secondary structure or DNA binding affinity was observed compared to protein purified without urea present. AmrZ was then passed over a MonoS cation exchange column, and eluted with a 0.1 M–1 M gradient of NaCl. Purity of the peak fractions was verified by SDS-PAGE, and fractions containing pure WT AmrZ were pooled. For crystallization experiments, AmrZ was dialyzed against 100 mM Bis-Tris pH 5.5, 100 mM NaCl, 2% glycerol, while for DNA binding assays, AmrZ was dialyzed against a buffer containing 100 mM Bis-Tris pH 6.5, 150 mM NaCl, 5% glycerol. WT AmrZ was then concentrated to 20 mg/ml for crystallization experiments, or 1 mg/ml for DNA binding assays, aliquoted, flash frozen in liquid nitrogen, and stored at −80°C. Concentration of WT AmrZ was measured using the BCA assay (Thermo Scientific) using a standard curve of lysozyme as a reference.

The Δ42 C-terminal truncation mutant of AmrZ was amplified from the *P. aeruginosa* strain PAO1 using the primers *amrZ_F* and *amrZ_Δ42_R* (5′-CGCCATCAGGATCCTCAAACACCGAGATTGTCTTG). Expression and purification of this protein was carried out using the procedures outlined for WT AmrZ.

### Crystallization of Δ42 AmrZ - amrZ1 complex

Crystallization trials of AmrZ were carried out by screening multiple AmrZ C-terminal deletion constructs against a library of double stranded DNA oligonucleotides containing the *amrZ1* binding site (Integrated DNA Technologies). Initial crystals were obtained only with the Δ42 AmrZ C-terminal truncation and an 18 bp oligonucleotide in a condition containing 6% PEG 8 K, 0.1 M MES pH 6.0, 0.1 M CaCl_2_, 0.1 M NaCl. For experimental phasing, selenomethionine (Se-Met) derivatized Δ42 AmrZ was prepared using published methods [50]. Purification of this protein was performed using the methods described above, with the only exception being the addition of 5 mM DTT in the final dialysis buffer. Crystals of the Se-Met Δ42 AmrZ - 18 bp *amrZ1* complex were obtained by mixing the protein and DNA in a 1∶1.5 molar ratio (810 µM AmrZ: 607.5 µM *amrZ1*) in the presence of 50 mM MgSO_4_. This complex was crystallized by the hanging drop vapor diffusion method at 25°C at a 1∶1 ratio with reservoir solution containing 3% PEG8K, 0.1 M MES pH 6.0, 0.15 M NaCl, and 2 mM TCEP pH 8.0. Crystals grew within 2–3 weeks and were soaked in a solution containing 20% 2-methyl-1,3 propanediol for cryo-protection before being frozen in liquid nitrogen for data collection.

### Data collection and refinement of the Δ42 AmrZ - amrZ1 structure

Diffraction data for crystals containing the Δ42 AmrZ: 18 bp *amrZ1* complex were collected on beamline X25 at the National Synchrotron Light Source (NSLS), Brookhaven National Labs. The dataset was collected at the selenium peak, with an X-ray wavelength of 0.9793 nm. Indexing, integration and scaling of the data were performed using HKL2000 program suite [51]. Phasing of the structure was performed using SAD methods with the program SOLVE [52], and density modification was performed using RESOLVE [53]. Manual model building was performed in Coot [54], and refinement was carried out using the programs REFMAC5 [55] within the CCP4 program suite [56], and CNS [57]. Data collection and refinement statistics are found in Table 1. The atomic coordinates and structure factors have been deposited in the Protein Data Bank under the PDB id 3QOQ.

### DNA binding measurements

Binding affinity of the various *amrZ1* and *algD* binding site mutants were performed using fluorescence anisotropy as previously described [30], [58]. In brief, increasing concentrations of WT AmrZ were incubated in a reaction (25 µl) containing 1 nM 22-mer 5′-6-carboxy-fluorescein (6-FAM) labeled DNA oligonucleotide (IDT) containing either the *amrZ1* or the *algD* sequences, 100 nM nonspecific DNA of random sequence, 100 µg/ml bovine serum albumin (BSA), 100 mM Bis-Tris pH 6.5, 150 mM NaCl, and 5% glycerol. DNA concentrations were kept 1 nM (<<K_d_) to ensure equilibrium measurements of binding constants. Anisotropy measurements were recorded at 25°C on a Safire2 microplate reader with a fluorescence polarization module (Tecan Group, Ltd.), using an excitation wavelength of 470 nm and an emission wavelength of 525 nm. Anisotropy data were scaled and normalized using Equation 1 below:
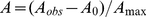
(1)In this equation, A_obs_ is the measured anisotropy value for each AmrZ concentration, A_0_ is the anisotropy of the unbound DNA, and A_max_ is the maximum anisotropy observed in each experiment. The dissociation constant (K_d_) was calculated by fitting the data to the equation for a single state binding model (Equation 2).
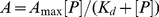
(2)Fitting of the data to Equation 2 was performed using SigmaPlot. Raw data and fits for AmrZ binding to each DNA sequence can be found in Figures S2 and S3, with the results from these experiments being presented in Tables 2 and 3. Results presented are the averages of four independent experiments.

### Calculation of protein and DNA parameters

The program CURVES+ [59] was used to measure the major and minor groove widths of the *amrZ1* DNA. The buried surface areas formed by protein-DNA interactions and by protein-protein interactions were measured using the programs AREAIMOL in the CCP4 suite [56] and PDBsum [60], respectively. Ramachandran statistics found in Table 1 were calculated with PDBsum [60]. Electrostatic surface representations of the protein and DNA were created by first generating a PQR file, which contains charge and radius information for each atom, with the program PDB2PQR [61], followed by visualization of the electrostatic surface using the APBS program [62]. All figures of structural representations were prepared using the program PyMol [63].

### amrZ promoter switching

The AmrZ binding site (ABS) (CCATTGGCCATTACCAGCCTCCC) in the *algD* promoter was replaced by the same-length *amrZ1* ABS (GTACTGGCAAAACGCCGGCACGC) from the *amrZ* promoter by site-directed mutagenesis [64]. Mutagenesis was achieved by primers *algD74* (GCGTGCCGGCGTTTTGCCAGTACATTACGCCGGAGATGGCATTTC) and *algD75* (GTACTGGCAAAACGCCGGCACGCGCCATTACATGCAAATTACGATTGC), together with flanking primers *algD65* (CCCCAAGCTTCTCTTTCGGCACGCCGAC) and *algD66* (CCGGGATCCCCGACATAGCCCAAACCAAAG). PCR products of *algD65/algD74* and *algD66/algD75* were denatured and hybridized. The products were used as the template for the second PCR, with primers *algD65* and *algD66*. With HindIII and BamHI sticky ends, the final PCR product was cloned into *HindIII* and *BamHI* double digested mini-CTX-*lacZ* transcriptional fusion vector [65], resulting in a new plasmid pBX8, which harbors modified *algD*::*lacZ* transcriptional fusion (*palgD_amrZ1_-lacZ*). The sequence of the *palgD_amrZ1_-lacZ* promoter was verified by PCR and sequencing.

### Construction of transcriptional fusion in *P. aeruginosa* chromosome

The plasmid pBX8 was transferred into *P. aeruginosa* FRD1 using *E. coli* strain SM10. The modified *palgD_amrZ1_-lacZ* transcriptional fusion was integrated at the *attB* site within the chromosome of FRD1 and FRD1 *ΔamrZ* [30], and the unnecessary portion of the fragment was removed by pFLP2 [66], resulting in FRD1 *palgD_amrZ1_-lacZ or FRD1 ΔamrZ palgD_amrZ1_-lacZ*, respectively.

### β-galactosidase assays

*P. aeruginosa* in mid-log phase were pelleted and washed with Z-buffer (110 mM Na_2_HPO_4_, 45 mM NaH_2_PO_4_, 10 mM KCl, 2 mM MgSO_4_, pH7.0). Cells were lysed through three rounds of fast freezing at −80°C then thawing at 37°C, followed by mild sonication. Samples were centrifuged at 18 k× g and 4°C for 10 min at 21,000× g. The supernatants were analyzed for β-galactosidase activity by mixing 10 µl of a sample supernatant with 80 µl of Z-buffer. To start the reaction 20 µl of 4 mg/ml orthonitrophenol was added. The color change in the reaction was monitored with time and reactions were stopped by addition of 40 µl 1 M Na_2_CO_3_ for reading. Wild type FRD1 *palgD*::*lacZ* transcriptional fusion was the positive control, and FRD1 *lacZ* with no promoter acted as the negative control. The absorbance at both 420 nm and 550 nm of each reaction solution was read in a Molecular Devices M5 microplate reader. Miller Units were calculated from different strains as outlined [67].

## Supporting Information

Figure S1 **Sequences of known AmrZ binding sites.** The two known AmrZ binding sites on the *amrZ* promoter leading to *amrZ* repression (*amrZ1* and *amrZ2*), and the one known binding site on the *algD* promoter, leading to activation of the alginate biosynthetic pathway are shown here. These sites have been determined experimentally through DNA footprinting experiments [6], [9], and share little consensus. The sequences are aligned based on the region of highest similarity. In the consensus above the sequences, uppercase nucleotides represent bases that are present in all three sequences, while lowercase nucleotides represent bases that are present in only two of the sequences.
(TIF)

Figure S2 **AmrZ -** ***amrZ1*** **binding data.** Fluorescence anisotropy was utilized to calculate the binding affinity for AmrZ to multiple sequences harboring mutations in the *amrZ1* binding site. Each data point is an average from four independent experiments, and the error bars are calculated from the standard deviation. Data was processed as described in the Materials and Methods section, and results from these data are shown in Table 2. (A) AmrZ: *WTamrZ1* (B) AmrZ: *GTCamrZ1* (C) AmrZ: *TTCamrZ1* (D) AmrZ: *CGCGamrZ1* (E) AmrZ: *TTC/GCamrZ1* (F) AmrZ: *ATATamrZ1* (G) AmrZ: *TTTTamrZ1* (H) AmrZ: *AATTamrZ1*.
(TIF)

Figure S3 **AmrZ -** ***algD*** **binding data.** To determine the AmrZ binding site on the *algD* promoter, multiple mutations to the *algD* DNA sequence were created and binding affinities between AmrZ and these sequences were determined using fluorescence anisotropy. Each data point is an average from four independent experiments, and the error bars are calculated from the standard deviation. Data from these experiments were processed as described in the Materials and Methods section, and results are shown in Table 3. (A) AmrZ: *WTalgD* (B) AmrZ: *m5algD* (C) AmrZ: *m6algD* (D) AmrZ: *m7algD* (E) AmrZ: *m8algD* (F) AmrZ: *m13algD* (G) AmrZ: *m14algD* (H) AmrZ: *m16algD* (I) AmrZ: *m17algD*.
(TIF)
