# Supplementary figures and images for: The Transcription Factor AmrZ Utilizes Multiple DNA Binding Modes to Recognize Activator and Repressor Sequences of Pseudomonas aeruginosa Virulence Genes
Source: PLoS Pathog. 2012 Apr 12;8(4):e1002648. doi: 10.1371/journal.ppat.1002648 (PMC3325190; doi:10.1371/journal.ppat.1002648)

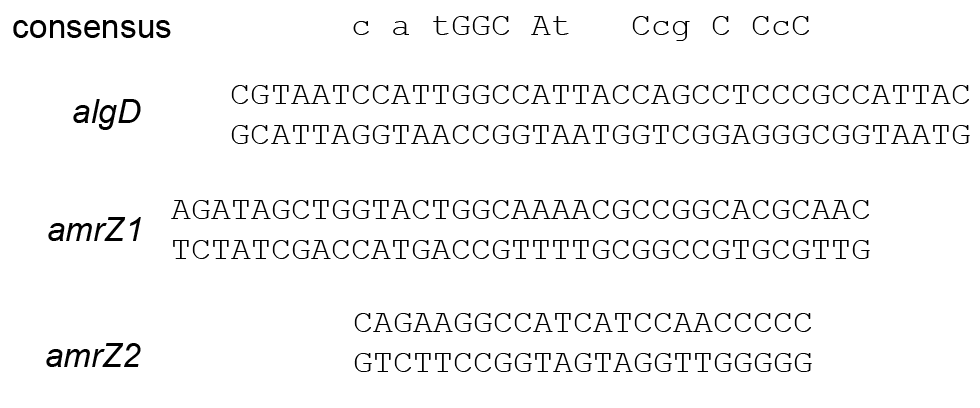

Supplement: Figure S1 — Sequences of known AmrZ binding sites. The two known AmrZ binding sites on the amrZ promoter leading to amrZ repression (amrZ1 and amrZ2), and the one known binding site on the algD promoter, leading to activation of the alginate biosynthetic pathway are shown here. These sites have been determined experimentally through DNA footprinting experiments [6], [9], and share little consensus. The sequences are aligned based on the region of highest similarity. In the consensus above the sequences, uppercase nucleotides represent bases that are present in all three sequences, while lowercase nucleotides represent bases that are present in only two of the sequences. (TIF) [file ppat.1002648.s001.tif]

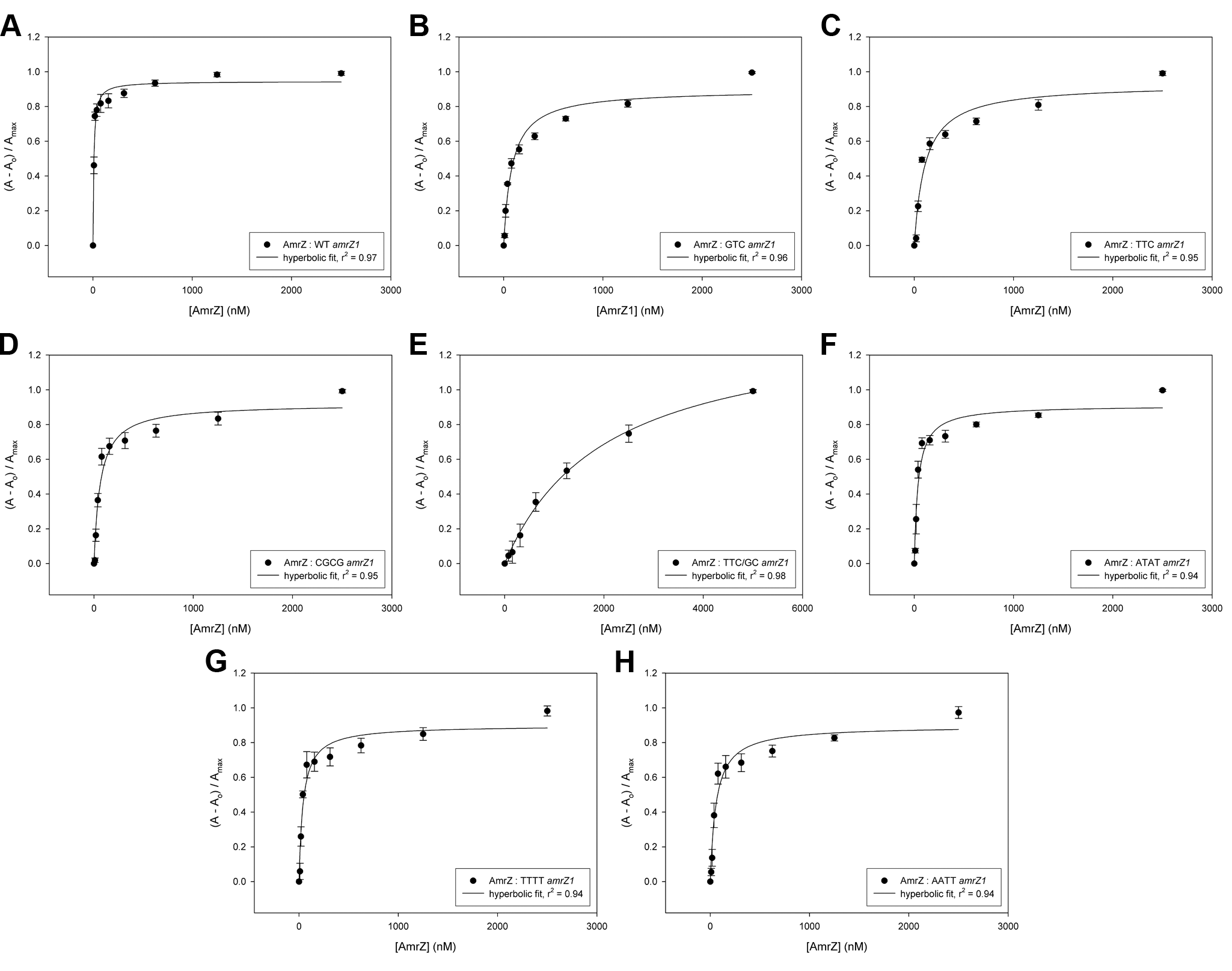

Supplement: Figure S2 — AmrZ - amrZ1 binding data. Fluorescence anisotropy was utilized to calculate the binding affinity for AmrZ to multiple sequences harboring mutations in the amrZ1 binding site. Each data point is an average from four independent experiments, and the error bars are calculated from the standard deviation. Data was processed as described in the Materials and Methods section, and results from these data are shown in Table 2. (A) AmrZ: WTamrZ1 (B) AmrZ: GTCamrZ1 (C) AmrZ: TTCamrZ1 (D) AmrZ: CGCGamrZ1 (E) AmrZ: TTC/GCamrZ1 (F) AmrZ: ATATamrZ1 (G) AmrZ: TTTTamrZ1 (H) AmrZ: AATTamrZ1. (TIF) [file ppat.1002648.s002.tif]

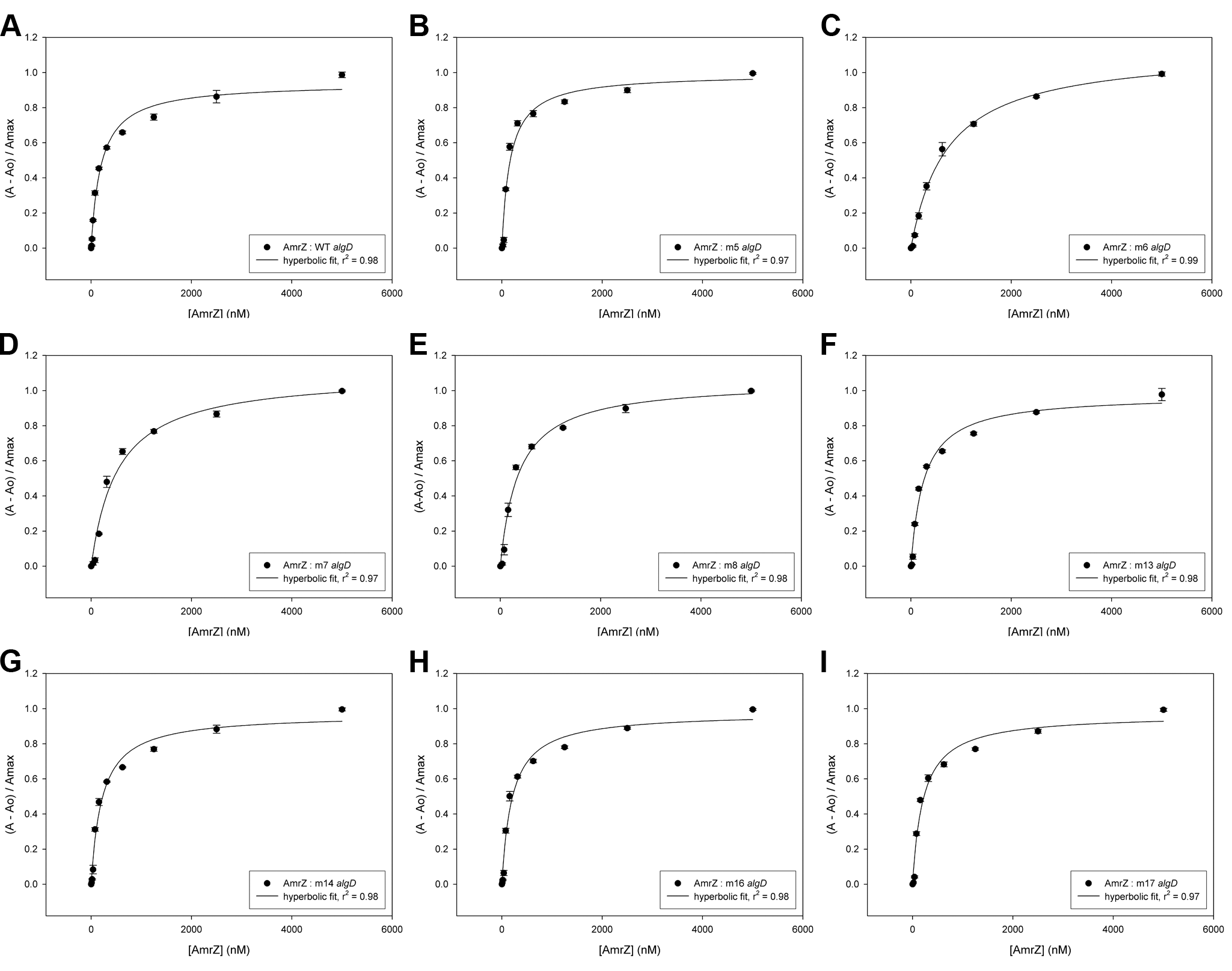

Supplement: Figure S3 — AmrZ - algD binding data. To determine the AmrZ binding site on the algD promoter, multiple mutations to the algD DNA sequence were created and binding affinities between AmrZ and these sequences were determined using fluorescence anisotropy. Each data point is an average from four independent experiments, and the error bars are calculated from the standard deviation. Data from these experiments were processed as described in the Materials and Methods section, and results are shown in Table 3. (A) AmrZ: WTalgD (B) AmrZ: m5algD (C) AmrZ: m6algD (D) AmrZ: m7algD (E) AmrZ: m8algD (F) AmrZ: m13algD (G) AmrZ: m14algD (H) AmrZ: m16algD (I) AmrZ: m17algD. (TIF) [file ppat.1002648.s003.tif]
